# Building Image Feature Extraction Using Data Mining Technology

**DOI:** 10.1155/2022/8006437

**Published:** 2022-04-13

**Authors:** Yi Deng, Chengyue Xing, Ling Cai

**Affiliations:** ^1^School of Architecture and Urban Planning, Guangzhou University, Guangzhou 510006, Guangdong, China; ^2^Guangdong Provincial Institute of Cultural Relics and Archaeology, Guangzhou 510075, Guangdong, China

## Abstract

At present, data mining technology is continuously researched in science and application. With the rapid development of remote sensing satellite industry, especially the launch of remote sensing satellites with high-resolution sensors, the amount of information obtained from remote sensing images has increased dramatically, which has largely promoted the application of remote sensing data in various industries. This technique mines useable information from less complete and accurate data while ensuring low program complexity. In order to determine the impact of data mining techniques on feature extraction of graphic images, this paper explores the relevant steps in the image recognition process, especially the image preenhancement and image extraction processes. This paper develops a preliminary set of relevant data and investigates two different extraction methods based on the availability or absence of nursing information. Aiming at the advantages and disadvantages of the two house extraction methods, this work discusses how to effectively integrate remote sensing data. It uses different data sources to describe different characteristics of buildings, analyzes and extracts effective information, and finally derives building information. The research results show that, using the SVM algorithm in data mining for image feature extraction, in the verified filtering window, the accuracy can be effectively improved by about 20%. Buildings are important objects in high-resolution remote sensing images, and their feature extraction and recognition technology is of great significance in many fields such as digital city construction, urban planning, and military reconnaissance.

## 1. Introduction

With the rapid development of the current city, the remote technology of urban buildings has attracted more and more attention of professionals. Synthetic Aperture Radar (SAR) and Pol Synthetic Aperture Radar (PolSAR) are widely used due to their daily characteristics and ability to store general weather forecast and multimedia information. This work uses data mining technology to study internal SAR and PolSAR imaging data mining technology, focusing on four aspects, retrieval analysis, attribution search, attribution allocation, and customization, and provides a complete remote city radar image system. An efficient and robust information retrieval system provides strong support for political planning, national economic construction, national security, and military equipment.

Data mining is the process of extracting hidden, unknown but useful information and knowledge from large amounts of flawed, noisy, chaotic, and random application data. Data mining can perform functions such as identification grouping, customization, prediction, cultural analysis, isolation analysis, and parallel analysis. How to accurately extract buildings has always been a current research hotspot. This paper proposes introducing the invariant moment algorithm into the feature extraction of buildings from high-resolution remote sensing images and verifying it through experiments.

This paper investigates and analyzes image processing technology, feature extraction and segmentation methods, and existing problems and introduces the development and status of imaging technology in detail. Data mining techniques are studied in detail and applied to the image recognition process. This paper introduces many common computing algorithms, compares the performance of different computing algorithms, and uses different computing algorithms at different levels and uses. It then briefly introduces and analyzes the advantages and disadvantages of different feature extraction algorithms.

## 2. Related Work

For data mining and feature extraction, domestic and foreign experts have done a lot of research. Angeli C. introduced two examples of using data mining techniques, namely, association rule mining and fuzzy representation. He found association rule mining to be a useful way to obtain reliable data on learners' use of simulations and their performance. His research illustrates how data mining can be used to advance the practice of educational software evaluation in the field of educational technology, addressing issues related to data privacy [1]. Zhang M. studied the definition of data mining technology, summarized the steps and methods of data mining technology, and analyzed its application in the badminton court tactical analysis system [2]. Divya S. proposed a framework for retrieving similar building plans under the example query paradigm. He proposed a new algorithm for extracting high-level semantic features from building floor plans. Later, fine-grained retrieval using weighted sum of features was proposed, during which one feature can be prioritized over other features [3]. Kuroda Y.'s research focused on the big data formed by images accumulated on the social media “Instagram” dedicated to image posting and mainly focused on the two following points. (1) big data visualization of museum images and (2) exploring big data analysis methods to help art museum planning. He made these findings not to show the subjectivity reflected in each image but to show some of the characteristics and impressions of the museum from the image data accumulated by visitors [4]. Yongjun detected unripe green fruits in citrus trees under natural light conditions. He developed three supervised classifiers, logistic regression, random forests, and support vector machines (SVMs), using texture features. Logistic regression, random forest, and SVM models have detection accuracies of 79%, 75%, and 86%, respectively. The algorithm he developed shows great potential for identifying immature green citrus for early yield estimation [5]. Edavoor P. J. proposed a new method to obtain a complete binary (low complexity) 6-tap orthogonally symmetric wavelet filter bank (FB) with near-perfect reconstruction. This is achieved by changing the PR conditions slightly to make the quadrature filter symmetric and obtain the full binary filter coefficients. His proposed wavelet FB provides comparable performance in image compression and achieves excellent performance in iris recognition systems (feature extraction) and OFDM [6]. Matsukawa G. introduced a low-power object recognition processor VLSI. The processor processes HDTV resolution video at 60 frames per second (fps) using an object recognition algorithm with sparse FIND features. Using this architectural design, he achieved 60 fps HDTV resolution video object recognition performance at 130 MHz operating frequency [7].

## 3. Building Image Feature Extraction Methods

### 3.1. Data Mining

**Figure 1:**
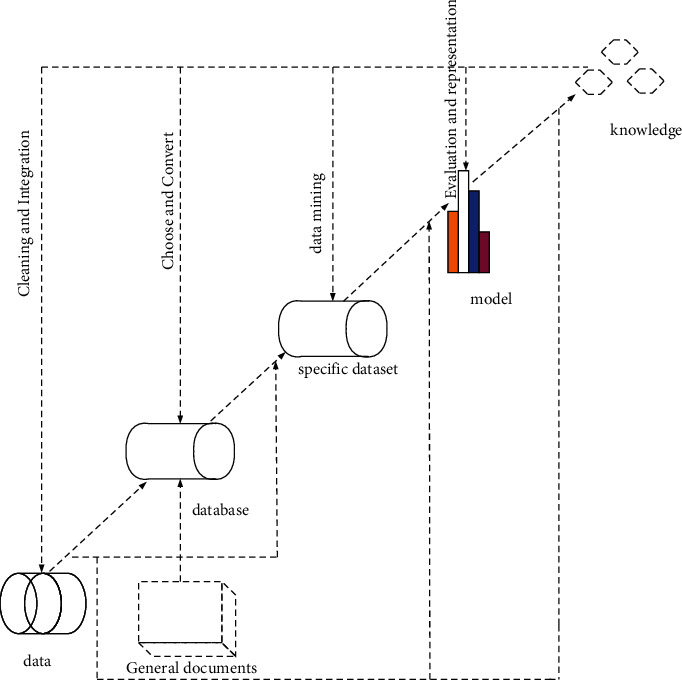
Basic steps of data mining.

Data mining is also used as a term for knowledge discovery (KDD), which is an important step in the knowledge discovery process [8–10]. The knowledge acquisition process follows these steps, as shown in Figure 1.

In the process of data mining, frequently used data systems have default information or wrong information. Data clearing is the process of clearing default data and erroneous data [11]. Data systems can identify extraneous data and process the extraneous data using mathematical calculations to arrive at the cause of the error. Data customization eliminates inconsistencies and default data in data and provides a more accurate data system for further data mining process to improve mining efficiency [12].

Data integration documents a more complete data system by transforming data in different formats into data in the same format. After cleaning the data and filling the default values, unnecessary features in the data system and features that are not relevant to the data mining step should be removed [13]. Data selection and data reduction are different from data systems. Data reduction is the removal of features, while data selection is the addition of feature data without affecting the results of cognitive research [14, 15].

The z-score transformation is usually used to transform the data into a normal distribution, because the general statistical analysis method assumes that the data obeys a normal distribution, and all models require the input data to be normally distributed. A z-score transformation needs to be applied. Data exchange is routine data processing. During data mining, the collected data can have different sources and different formats. The data must therefore be balanced to obtain a simple data format for processing in the data mining step [16]. The data mining step allows intelligent analytical methods to extract data and manipulate procedures. According to the user's needs, it selects the appropriate algorithm, uses the selected algorithm to analyze the data, obtains the desired knowledge, and presents the results through visualization and other means [17].

Data mining is mainly based on aggregation algorithm, which can be subdivided into partition method, process state method, density source method, and grid source method. Among them, a feature of the hierarchical approach is the multirow customization of the database, which is suitable for high-resolution data [18–20]. In a cluster-based clustering system, the uncertainty of the weight parameters has a significant impact on the clustering effect. Although the weight of each cluster is uneven, the variation is large, and the clustering effect is poor. The grid-based loading method is fast loading, which is fast, but the quality and accuracy are not enough [21]. The partition-based loading method is simple and efficient and can handle large data systems without worrying about noise. In view of the large amount of data using household energy and the characteristics of some data silencing, the article chooses a group-based analysis method [22]. By analyzing the physical location of secret files and directories, all information is erased bit by bit, and random characters or custom characters can be used to fill multiple times to ensure that the storage medium data is overwritten multiple times to achieve the purpose of unrecoverable file data. This paper analyzes the current research progress on building extraction from remote sensing images. From the literature and research status, it can be seen that the invariant moment algorithm has a good identification function in the matching and retrieval of digital images and databases.

The grouping algorithm divides data objects into multiple parts according to the characteristics of the data to reduce the external similarity of each category and increase the internal similarity of each element. While both grouping and adaptation are data isolation, grouping is an unmanaged learning process, while isolation is a managed training process [23]. The isolation algorithm must first understand the structural characteristics of the data and classify according to the characteristics of the data structure and at the same time integrate the algorithm to find the characteristics of the data structure. Therefore, in many data mining processes, grouping is also used as part of data mining to record data structure elements. The study of loading algorithms involves several loading algorithms, including distribution method, location method, weight source method, grid source method, and model source method [24]. Image processing is the technique of analyzing images with a computer to achieve the desired results. Image processing generally refers to digital image processing. Digital image refers to a large two-dimensional array obtained by shooting with industrial cameras, video cameras, scanners, and other equipment. The elements of the array are called pixels, and their values are called gray values.

### 3.2. SVM Algorithm

The advantage of SVM is to solve small sample, nonlinear, and high-dimensional regression and binary classification problems. Small sample means that the number of samples required by SVM is relatively small compared to the complexity of the problem. Support vector machine was first proposed in 1995, which is based on statistical theory and was originally designed for binary classification problems. The algorithm not only introduces the concept of structural risk but also adopts the idea of kernel mapping. SVM algorithm shows many advantages in solving small samples, nonlinear data, and high-dimensional spatial pattern recognition.

The binary classification problem is also called binary classification. In the training data sample space, the sample data categories are divided into two categories. The purpose of the binary classification problem is to find a classifier that can successfully separate the two types of data.

**Figure 2:**
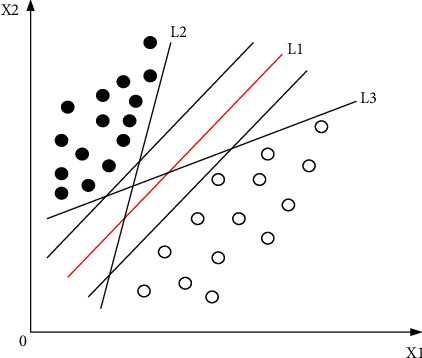
Schematic diagram of binary classification in two-dimensional space.

Figure 2 shows the binary classification problem in a two-dimensional space, and the solid and hollow points represent two types of training data. In two-dimensional space, if a straight line can separate two types of data, such as straight lines L1, L2, and L3 in the figure, these data are said to be linearly separable; otherwise they are nonlinearly separable. The actual discriminant function can be expressed as

(1) $$\begin{array}{c} f\left(x\right)=\mathrm{sgn}\left(g\left(x\right)\right), \end{array}$$

where *f*(*x*) is the value of the discriminant function. The distance from a point in space to the hyperplane can be written as

(2) $$\begin{array}{c} \delta =\frac{\left|g\left(x\right)\right|}{\left\|w\right\|}=\frac{\left|w^{t}x+b\right|}{\left\|w\right\|}, \end{array}$$

where *δ* is called the geometric interval and the geometric interval represents the Euclidean distance from the sample point in the space to the hyperplane.

Using *y* to represent the sample category, it is obvious that the sample points meet the following conditions:

(3) $$\begin{array}{c} \left\{\begin{array}{c} w^{t}x+b\geq +1,y=+1,\\ w^{t}x+b\leq +1,y=-1. \end{array}\right. \end{array}$$

In order to determine the optimal classification hyperplane, it is necessary to solve conditions *w* and *b* under the premise of the largest geometric interval; namely,

(4) $$\begin{array}{c} \left\{\begin{array}{c} \begin{array}{cc} \underset{w,b}{\max,} & \frac{2}{\left\|w\right\|}, \end{array}\\ \begin{array}{cc} s.t., & y\left(wtx+b\right)\geq 1,i=1,2,...,n. \end{array} \end{array}\right. \end{array}$$

In order to facilitate the calculation of the deduction process such as derivation in the future, it can be rewritten as

(5) $$\begin{array}{c} \left\{\begin{array}{c} \begin{array}{cc} \underset{w,b}{\min,} & \frac{1}{2}{\left\|w\right\|}^{2}, \end{array}\\ \begin{array}{cc} s.t., & y\left(wtx+b\right)\geq 1,i=1,2,...,n. \end{array} \end{array}\right. \end{array}$$

The above formula is the basic type of SVM. Its objective function is a quadratic function of *w*, and the feasible region is a convex set, which is a convex quadratic programming problem. In order to solve efficiently, the Lagrange multiplier is introduced to obtain

(6) $$\begin{array}{c} L\left(w,b,a\right)=\frac{1}{2}{\left\|w\right\|}^{2}+\overset{n}{\underset{i=1}{\sum }}a\left(1-y\left(w^{t}x+b\right)\right), \end{array}$$

where the vector is *a*=(*a*_1_, *a*_2_, ..., *a*_*n*_) and the partial derivatives of *w* and *b* are equal to 0; there are

(7) $$\begin{array}{c} w=\overset{n}{\underset{i=1}{\sum }}a_{i}y_{i}x_{i}, \end{array}$$

(8) $$\begin{array}{c} 0=\overset{n}{\underset{i=1}{\sum }}a_{i}y{}_{i}. \end{array}$$

The following can be obtained by substituting the formula into its dual problem:

(9) $$\begin{array}{c} \left\{\begin{array}{cc} \underset{a}{\max}, & \overset{n}{\underset{i=1}{\sum }}a_{i}-\frac{1}{2}\overset{n}{\underset{i=1}{\sum }}\overset{n}{\underset{j=1}{\sum }}a_{i}a_{j}y_{i}y_{j}x^{t},\\ s.t., & \overset{n}{\underset{i=1}{\sum }}a_{i}u_{i}=0,a_{i}\geq 0,i=1,2,..,n. \end{array}\right. \end{array}$$

In the above formula, all are known quantities except *a*, and the discriminant function can be obtained by solving *w* and b:

(10) $$\begin{array}{c} f\left(x\right)=\mathrm{sgn}\left(g\left(x\right)\right)=\mathrm{sgn}\left(\overset{n}{\underset{i=1}{\sum }}a_{i}y_{i}x_{i}^{t}+b\right). \end{array}$$

With the development of remote sensing science and technology, the accuracy of remote sensing data is getting higher and higher, the acquisition speed is getting faster and faster, and the amount of data acquired is getting larger and larger, but the amount of information acquired by a single sensor is limited, and it is often difficult to meet the needs of applications. In practical applications, the training data is often nonlinearly separable. A linear hyperplane cannot completely separate the sample points correctly. At this time, in order to obtain a linear hyperplane, the points in the sample space can be mapped to a higher-dimensional space.

The classification function that maps to a higher-dimensional space can be rewritten as

(11) $$\begin{array}{c} g\left(x\right)=w^{t}\theta \left(x\right)+b. \end{array}$$

The above formula can be rewritten as

(12) $$\begin{array}{c} \left\{\begin{array}{cc} \underset{a}{\max}, & \overset{n}{\underset{i=1}{\sum }}a-\frac{1}{2}\overset{n}{\underset{i=1}{\sum }}\overset{n}{\underset{j=1}{\sum }}a_{i}a_{j}y_{i}y_{j},\\ s.t., & \overset{n}{\underset{i=1}{\sum }}a_{i}y_{i}=0,ai\geq 1,2,...,n. \end{array}\right. \end{array}$$

There exists a function that accepts input values in a low-dimensional space. It calculates the inner product value of the high-dimensional space mapped to

(13) $$\begin{array}{c} K\left(x_{i},x_{j}\right)=\theta \left(x_{i}^{t}\right)\theta \left(x_{i}\right). \end{array}$$

This function *K* is called the kernel function.

For nonlinearly separable training vectors, it can be extended by modifying the formula

(14) $$\begin{array}{c} \begin{array}{cc} \left(W*x_{i}\right)+b\geq 1-\gamma & y_{i}=1 \end{array}, \end{array}$$

(15) $$\begin{array}{c} \begin{array}{cc} \left(W*x_{i}\right)+b\leq -1+\gamma & y_{i}=-1 \end{array}. \end{array}$$

### 3.3. Remote Sensing Technology Feature Extraction

**Figure 3:**
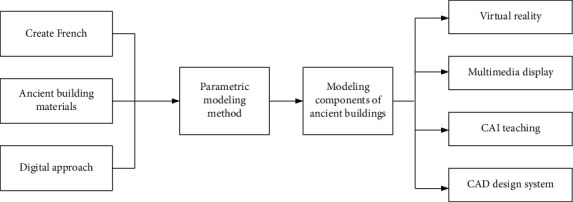
System implementation process.

Airborne LiDAR systems acquire spatial information by acquiring high-precision 3D coordinates of ground targets. However, the airborne LiDAR cannot obtain the attribute information of the ground object surface from the point cloud information. This leads to the inability to construct the structure of the ground object based on the point cloud information, which increases the difficulty of extracting the topological information of the ground object. At present, airborne radar technology has accumulated rich experience in data acquisition and has relatively mature point cloud acquisition technology. However, the processing of airborne radar data is still in the research and development stage, and the postprocessing technology of the data is still relatively lagging behind. Most of the current point cloud data postprocessing algorithms have certain defects, and some algorithms with better performance have harsh conditions and poor applicability. The biggest problem that limits the development of point cloud data postprocessing technology is the filtering of point cloud data. Point data filtering is a fundamental and very important step in postprocessing of airborne LiDAR data. The pros and cons of point cloud filtering accuracy directly image the subsequent point cloud classification, object recognition, DEM generation, and 3D reconstruction of buildings. The realization process of the 3D reconstruction system is shown in Figure 3.

Morphological algorithms applied to image processing are based on nonlinear set theory. The biggest advantage of morphological theory is that it introduces the concept of “structural elements.” The basic idea is to use a set of known structural elements to perform correlation operations with images. According to the set judgment rules, it judges the attributes of the pixels in the coverage area of the structural elements and verifies the validity of the filled structural elements. Sequential structure is the simplest program structure and the most commonly used program structure, as long as the corresponding statements are written in the order in which the problem is solved, and its execution order is top-down and sequentially executed.

**Figure 4:**
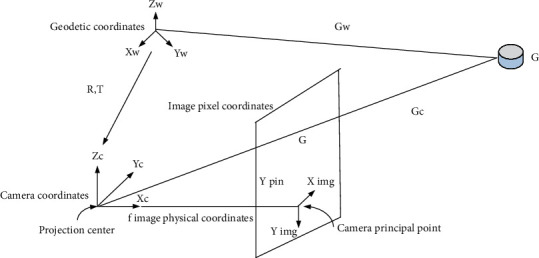
Strict sensor model imaging principle.

As shown in Figure 4, according to the transformation relationship between the geodetic coordinates, camera coordinates, image physical coordinates, and image pixel coordinates during the imaging process of the optical sensor, the coordinates of the target point are obtained and the structural elements have various geometric characteristics.

**Figure 5:**
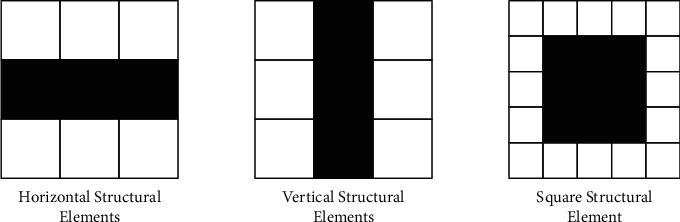
3 ^*∗*^3 structural elements.

The combination of elements of different structures will have different binding property characteristics. In practical applications, it is necessary to appropriately select structural elements to perform related operations according to the image characteristics to be processed. When processing images, we require a rectangular array of structuring elements, which is achieved by adding the smallest possible number of background elements to form a rectangular array. According to the geometrical characteristics of structural elements, structural elements can be divided into horizontal structural elements, vertical structural elements, square structural elements, diamond structural elements, and other types. The following are commonly used structural elements, as shown in Figure 5.

According to the transformation relationship between O geodetic coordinates, camera coordinates, image physical coordinates, and image pixel coordinates during the imaging process of the optical sensor, the coordinates of the target point are got, and the calculation method is

(16) $$\begin{array}{c} G_{w}=\left(X_{w},Y_{w},Z_{w}\right). \end{array}$$

The camera coordinates are

(17) $$\begin{array}{c} G_{c}=\left(X_{c},Y_{c},Z_{c}\right). \end{array}$$

There exists the following relationship between them:

(18) $$\begin{array}{c} Gc={\left[Xc,Yc,Zc\right]}^{T}=R\cdot Gw-T. \end{array}$$

It establishes the connection between the physical coordinates *G* of the image and the camera coordinates.

(19) $$\begin{array}{c} \left\{\begin{array}{c} x_{img}=f\frac{X_{c}}{Z_{c}}\\ y_{img}=f\frac{Y_{c}}{Z_{c}} \end{array}\right. \end{array}$$

We can get

(20) $$\begin{array}{c} \left\{\begin{array}{c} x_{img}=f\frac{r_{11}\left(Xw-Xs\right)+r_{12}\left(Yw-Ys\right)+r_{13}\left(Zw-Zs\right)}{r_{31}\left(Xw-Xs\right)+r_{32}\left(Yw-Ys\right)+r_{33}\left(Zw-Zs\right)},\\ y_{img}=f\frac{r_{21}\left(Xw-Xs\right)+r_{22}\left(Yw-Ys\right)+r_{23}\left(Zw-Zs\right)}{r_{31}\left(Xw-Xs\right)+r_{32}\left(Yw-Ys\right)+r_{33}\left(Zw-Zs\right)} \end{array}\right.. \end{array}$$

This is not the final strictly sensor imaging model yet. On this basis, the relationship between image pixel coordinates and image coordinates also needs to be considered, as shown in the following formula:

(21) $$\begin{array}{c} \left\{\begin{array}{c} x_{pix}=\frac{x_{img}}{S_{x}}+o_{x},\\ y_{pix}=\frac{y_{img}}{S_{y}}+o_{y}. \end{array}\right. \end{array}$$

The strict sensor model defined by the collinear formula is rigorous in theory, and the relevant parameters are directly related to the physical quantities of the imaging sensor, which has distinct significance.

Compared with point cloud classification processing, the technology of digital image processing is quite mature. Image preprocessing technology, image segmentation, image compression, image restoration, and reconstruction technology have been very mature, and some new wavelet processing, morphological processing, and neural network technology have also achieved good results. Image processing technology has been successfully used in remote sensing, printing, medical imaging, and other fields.

The single-polarization SAR image can complete the urban scene classification, but, because of the limited information of the target scattering mechanism, it cannot effectively distinguish the buildings with different orientations. The advantage of PolSAR images is that the scattering mechanism of different types of objects can be characterized by using the polarization target decomposition method. It in turn can utilize the target scattering power to achieve urban classification of PolSAR images. Buildings with different radar orientations have different scattering mechanisms on PolSAR images. The multiphase polarization target path fault based on the scattering model proposed in this paper can illustrate the diffusion pattern of buildings under different radar azimuth conditions, which can be used to judge the particularity of cities. In view of the superpixels proposed in this paper which can help improve the classification accuracy of remote sensing images, the next step is to use superpixels to replace single pixels to achieve superpixel-level classification.

However, compared with the point cloud data, the image contains less information, and there is no coordinate information of the ground objects. At the same time, with the continuous advancement of technology, high-resolution image acquisition has become easier. High-resolution images bring not only more information but also more interference information, such as the green belts on both sides of the road, the interference of vehicles, and pedestrians on the road. Secondly, in most cases, the images obtained by photogrammetry are nonorthophoto images. This means that what we see is not what we get. That is, there is a certain deviation between the objects displayed on the image and their real geographic locations. This is mainly caused by the certain flight attitude of the aircraft and many other uncertain factors when collecting data. Therefore, image preprocessing needs to perform image correction, and there will inevitably be errors in the process of image correction, which will improve the accuracy of later feature extraction. The collection of point cloud data, due to the strict flight requirements and the use of GPS/inertial navigation system, can detect and record the flight attitude of the aircraft in real time. It can obtain high-precision coordinate information by using the supporting solution software. That is, “what you see is what you get,” and the point cloud reflects the real information of the ground objects.

### 3.4. Image Processing

In image recognition technology, the image quality directly affects the accuracy of the recognition algorithm. Therefore, imaging technology plays an important role in the entire image recognition process. Its main purpose is to remove trivial factors in images, add useful information, and make data as simple as possible to improve the reliability and accuracy of operations. Commonly used basic imaging algorithms include sampling algorithm, processing algorithm, and convergence algorithm. In order to achieve good results in practical applications, the above algorithms have their own characteristics in implementation methods. Imaging is the imaging technology of biological samples, which can be roughly divided into tissue imaging and cellular and molecular microscopy according to the size of the sample. These generally require the development of optical technology in conjunction with the characteristics of biological samples, and a few use wave properties other than light, such as nuclear magnetic resonance and ultrasound.

Due to various environmental factors, images can contain a lot of unwanted information. The filtering algorithm is to multiply the element parameter by the corresponding position, take the sliding window as the center, and take the corresponding value. Imaging algorithms have boundary issues such as performance and convergence. This means that when the limit cannot be improved, the further designed limit is treated separately. The general application scenarios of this algorithm are as follows. The size of the actual image cannot meet the constraints of the processing algorithm where the input panel is a multiple of the window size. The self-processing algorithm is computed by moving the sash backwards. Therefore, there will be some border elements without pixel values around them, which cannot be calculated by integration and require additional processing. By detecting the extreme point of the parameter in the parameter space, the curve corresponding to the parameter in the space domain is determined, so as to extract the regular curve in the image.

Among them, the sampling algorithm is divided into an upsampling algorithm and a downsampling algorithm. Bottom sampling refers to the process of compressing the original image to reduce the image to a certain factor, depending on the actual application scenario. The basic rule is to take a small image after calculation based on the local data in the image input panel. The upgrade process is to enlarge the image below to the original size. The whole process and parameters will be determined according to the actual application. However, the kernel process is implemented by computing the correlation of the local data of the original image with the parameters of the table. The goal of image preparation is to minimize the image when both the input and output are bright images. Currently, image processing matrices are commonly used to represent the value of each pixel in an image. The main purpose of preprocessing is to improve image data and provide technical support for subsequent image processing. The image processing material discussed in this article is part of image enhancement, where similar parallel processing algorithms are used to enhance visual effects and replace image features.

The experimental data must be preprocessed before image capability can be removed. In the process of identifying a building, it is important to highlight the structure and corners of the building. Therefore, grayscale and filtering functions are usually performed to weaken the junk information in the image and highlight the valuable information. Since this process basically computes the correlation between the pixel table and the input image, it has good parallelism.

## 4. Experimental Design and Result Analysis

Before the experiment, the parameters in the algorithm need to be set. When using the SVM algorithm for three classifications, the Libsvm toolbox is used for experiments, and the RBF kernel function is applied. It uses the svm_cross_validation function and the grid_search function to optimize parameters and obtain the optimal parameters. The cost matrix of the two-state decision problem is shown in Table 1.

**Figure 6:**
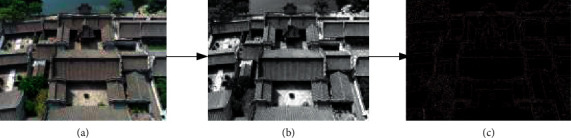
Building image edge detection: (a) original image, (b) image grayscale, and (c) edge detection image.

It takes an ancient building as an example. First, the building is processed in grayscale, and then edge detection is performed with the Log operator to extract features. The result is shown in Figure 6.

**Figure 7:**
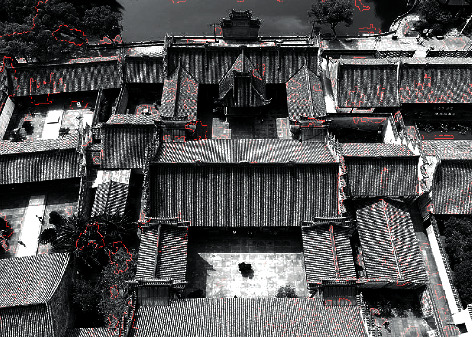
Feature extraction results.

The extracted features are marked, and the result is shown in Figure 7.

**Figure 8:**
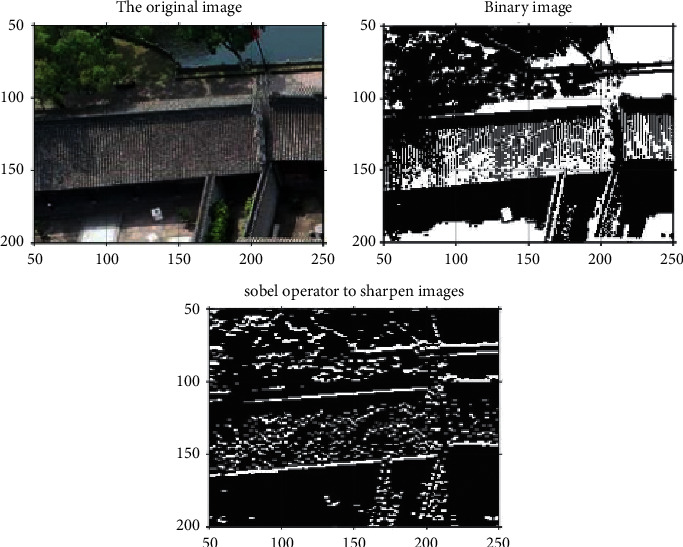
Image segmentation optimization.

It selects a corner of the building and processes its features, and the result is shown in Figure 8.

It can be seen that, after image segmentation and threshold segmentation optimization, the results of building removal are compared with the results obtained by the previous single method, the results of total building removal are significantly improved, and the basic layout and shape of buildings are kept well. However, the data also showed that there were still some components that were mistakenly removed and omitted. The main reason for the disappearance is that when the SVM algorithm performs image segmentation, the area distribution on the roofs of some buildings with opposite textures is different. Some of this is due to the angle of incidence of the light, and the roof is partially obscured, and some roofs have uneven and uniform levels of texture due to various objects like skylights. Drawing on the current research status of building image classification and the development of feature extraction technology, color features, texture features, and shape features are established as the indicators of building image classification.

**Figure 9:**
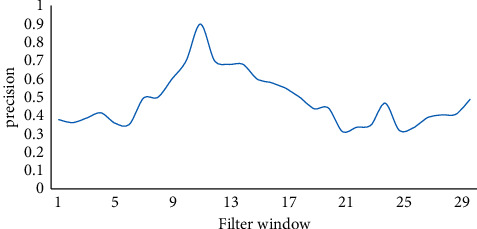
Building extraction accuracy.

As shown in Figure 9, with the continuous increase of the filter window, the accuracy of the building exit shows a trend of first increasing and then decreasing. It can effectively improve the influence of small structural differences on the final output accuracy. The accuracy of suitable window extraction can be effectively improved by about 20%.

**Figure 10:**
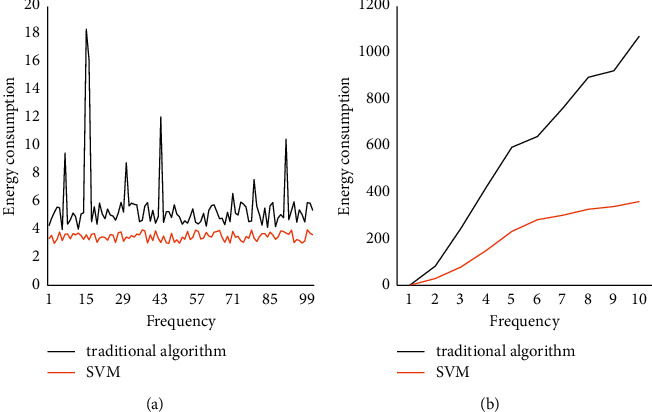
Energy consumption comparison of different methods. (a) Unit energy consumption. (b) Overall energy consumption.

For the cross-scattering components of the data and the polarization target decomposition results obtained by different methods. We compared the results of different mining methods in building image processing, and the energy consumption results of different methods are shown in Figure 10.

It can be seen from Figure 10 that, under different bowl fern methods, the energy consumption required for feature extraction of buildings is also different. The unit energy consumption of the traditional extraction method is about 6, and, with the increase of extraction times, the unit energy consumption will be too high. In the SVM algorithm used in this paper, the unit energy consumption is stable at about 3, which is much better than the traditional method.

We make statistics on the percentage of different scattering components when the buildings are located in different areas, and the results are shown in Tables 2 and 3.

**Figure 11:**
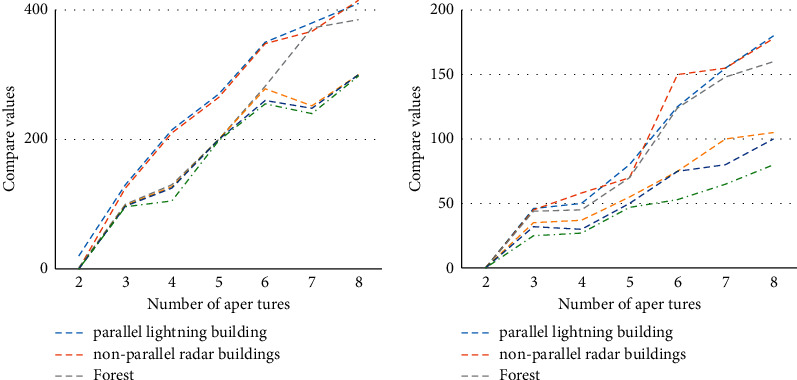
Aperture decomposition and the effect of reflection asymmetry on building detection.

It discusses the influence of aperture decomposition and reflection asymmetry on building detection, sets the number of image subapertures to 2, 3, 4, 5, 6, 7, and 8, and repeats the traditional detection algorithm and SVM algorithm. The result is shown in Figure 11.

It can be seen that the forest and the two different types of buildings are always mixed together and indistinguishable, and the log-likelihood ratios of the three types of targets of road, small target, and bare ground are also very similar under different subapertures. This is because the nonstationarity of roads and small targets is weak under limited subapertures, and if the number of subapertures is increased, the image resolution will become lower, resulting in loss of target information.

## 5. Discussion

Although the morphological algorithm can filter out the noise on the image, the original image has too much noise. Its use of morphological filtering algorithm will destroy edge information to a certain extent. Such noise can be filtered out by performing multiple ring morphological algorithms. We used the circular complementary morphological filtering algorithm twice, using a 5 × 5 circular template and a 3 × 3 square template for the complementary template. The number of iterations used by the algorithm depends on the actual image noise. Larger images and more noisy images can increase the number of iterations. However, too many iterative calculations will destroy the edge information to a certain extent, so it is necessary to synthesize the whole process of processing to reasonably select the number of iterations.

Taking the actual survey area as an example, this paper elaborates the process of extracting buildings from LiDAR images using the improved morphological filtering algorithm described in this paper. Judging from the extraction effect of LiDAR point cloud image in the survey area in the actual project, the extraction framework is constructed reasonably.

The method described in this paper has high generality, and the effect of building edge detection is ideal. Based on the concept of multisource data processing, this paper applies imaging technology to the LiDAR cloud computing platform. In this work, traditional morphological processing is continued based on the properties of lidar point cloud data and the geometric features of lidar images. The usefulness and reliability of the method are proved by tests:

① According to scientific principles, taking into account the geometric characteristics of the building and the characteristics of the building space cloud, this work developed a custom automatic algorithm. It proposes a ring structure algorithm that enhances the smooth running of its surface. Experiments show that the algorithm has a good effect in removing the additive noise of the image and protecting the edge of the image.
② Due to the variability and inaccuracy of the distribution of airborne LiDAR lasers, as well as the influence of environmental factors during operation, the detected surface density is not uniform, resulting in inconsistent LiDAR images. Aiming at the complete hole problem, this work proposes a model algorithm and a two-dimensional object-based filling algorithm according to a scientific method, which can effectively protect the edge information of the image.
③ Compared with the traditional image processing algorithm, the SVM algorithm used in this paper has a great improvement in image clarity, edge output accuracy, and image quality. The image distortion is low, which confirms the reliability, applicability, and usability of its advanced algorithm.

The classification of urban buildings from radar remote sensing images is one of the important applications in the field of urban remote sensing. It is of great significance to urban planning, dynamic monitoring of buildings, and urban disaster monitoring. Since the radar is a side-view imaging, the azimuth of the building relative to the radar directly affects its scattering characteristics on the radar image. Buildings with different orientations are also of great significance in actual urban planning.

## 6. Conclusion

In this paper, a building feature extraction method based on SVM algorithm is proposed. First, it combines high-resolution spectral data with DSM data. Second, it extracts the underlying features of the merged data, including local neighborhood features and structural features. Next, the high-level semantic features of buildings are summarized through SVM, and the high-level semantic features and low-level initial features are combined, and the buildings are rederived using SVM. Finally, morphological filters are used to improve the extraction results. The results show that the building derivation algorithm studied in this paper has a strong application value for the reconstruction of ground objects. It can be considered that the key areas of the image are divided into blocks to extract key features, so that the acquisition of features is more accurate and complete, thereby providing guarantee for subsequent accurate classification.

## Figures and Tables

**Table 1:** Two-state decision problem cost matrix.

Decision action	Objective state of the entity	
	Normal (*X*)	Abnormal (−*X*)
Normal	*γ*_*pp*_	*γ*_*pn*_
Uncertain	*γ*_*BP*_	*γ*_*BN*_
Abnormal	*γ*_*NP*_	*γ*_*NN*_

**Table 2:** Scattering component ratios (overall) obtained by different methods.

Scattering component	Scatter component percentage		
	SVM	BP neural network	Bayesian
Surface scattering	34.5	34.1	36.3
Even scattering	55.9	55.2	51.7
Volume scattering	6.3	5.8	7.3
Spiral scattering	2.8	2.5	2.1

**Table 3:** Scattering component ratios (corners) obtained by different methods.

Scattering component	Scatter component percentage		
	SVM	BP neural network	Bayesian
Surface scattering	2.7	8.2	0.8
Even scattering	5.2	8.8	2.5
Volume scattering	85.6	72.3	84.1
Spiral scattering	7.9	7.5	7.3

## Data Availability

The data that support the findings of this study are available from the corresponding author upon reasonable request.
